# Effect of capacity building interventions on classroom teacher and early childhood educator perceived capabilities, knowledge, and attitudes relating to physical activity and fundamental movement skills: a systematic review and meta-analysis

**DOI:** 10.1186/s12889-024-18907-x

**Published:** 2024-05-27

**Authors:** Matthew Bourke, Ameena Haddara, Aidan Loh, Kendall A Saravanamuttoo, Brianne A Bruijns, Patricia Tucker

**Affiliations:** 1 School of Occupational Therapy, Faculty of Health Sciences, Western University, Elborn College, 1201 Western Road, London, ON N6G 1H1 Canada; 2 Children’s Health Research Institute, 800 Commissioners Road East, London, ON N6C 2V5 Canada; 3 Health and Wellbeing Centre for Research Innovation, School of Human Movement and Nutrition Sciences, The University of Queensland, Brisbane, Australia

**Keywords:** Physical activity, Fundamental movement skills, Capacity building, Training, Teachers, Early childhood educators

## Abstract

**Background:**

Capacity building may play an important role in improving classroom teachers’ and early childhood educators’ (ECE) capacity to implement physical activity and FMS interventions. Capacity building is the development of knowledge, skills, and structures to improve the capability of individuals and organisations to achieve effective health promotion. This review aimed to determine the efficacy of capacity building interventions on teachers’ and ECEs’ perceived capabilities, knowledge, and attitudes relating to physical activity and fundamental movement skills.

**Methods:**

An exhaustive literature search of six electronic databases was conducted. Controlled, single-group pre-post studies were included if they measured the effect of a capacity building intervention on in-service or pre-service classroom teachers’ (primary or secondary) or ECEs’ physical activity or fundamental skills related perceived capabilities, knowledge, or attitudes. The effects of interventions were synthesised using random effects meta-analysis. Subgroup analysis and meta-regression was conducted to determine if the effects differed based on study design, type of teacher (ECE vs. primary school), or teacher level (pre-service vs. in-service).

**Results:**

A total of 22 studies reporting on 25 unique samples were included in the meta-analyses. Only studies reporting on ECEs and primary school teachers were identified. Interventions most commonly included training/professional development, resources and toolkits, communities of practice, mentorships, and ongoing support. Results showed that capacity building interventions significantly improved teachers’ and ECEs’ perceived capabilities (g = 0.614, 95% CI = 0.442, 0.786), knowledge (g = 0.792 95% CI = 0.459, 1.125), and attitudes (g = 0.376 95% CI = 0.181, 0.571). The effects did not differ significantly as a function of any of the moderators examined.

**Conclusion:**

Findings from this review provide strong support that capacity building interventions are efficacious at improving teachers’ and ECEs’ perceived capabilities, knowledge, and attitudes related to promoting physical activity and teaching fundamental movement skills. Pre-service teachers and ECEs should be provided training in physical activity and fundamental movement skills as part of their degrees, and continual professional development and capacity building should be offered to in-service teachers and ECEs to promote physical activity and fundamental movement skills in children.

**Supplementary Information:**

The online version contains supplementary material available at 10.1186/s12889-024-18907-x.

## Registration

CRD42022330335.

## Background

There are a multitude of physical, mental, cognitive, and social health benefits associated with participation in physical activity for children and adolescents [1–3]. Additionally, being physically active during childhood and adolescence can promote physical literacy and contribute to lifelong engagement in physical activities [4]. Further, developing fundamental movement skills (FMS), a specific set of gross motor skills encompassing locomotor (e.g., running, jumping) and object control (e.g., throwing, catching) skills, in children may be the building blocks for children to engage in more complex movement behaviours and promote the engagement in multiple different types of physical activities [5, 6]. There is initial but growing evidence showing that cultivating FMS and engaging in more physical activity can improve a range of outcomes relevant to the classroom including executive functioning and academic performance [7, 8].

Children spend the majority of their waking time in educational settings (including preschools, childcare, elementary/middle schools, and secondary schools). Consequently, these settings have been identified as an ideal environment to promote engagement in physical activity and teach FMS [9–11]. Although secondary schools and some elementary schools have specialist Physical Education (PE) teachers, who have the skills and training to ensure children are physically active while at school and to teach children FMS, on average, schools do not provide children with at least 150 min of PE each week [12]. Additionally, most elementary schools do not have a specialised PE teacher [12, 13] and for children in preschool/childcare centres, early childhood educators (ECEs) rarely receive specialised PE training [14, 15]. Therefore, promoting engagement in physical activity and FMS development in educational settings cannot be considered solely in the context of formalised PE programs and only a priority for specialist PE teachers [16].

Outside of formalised PE programs, classroom teachers and ECEs play an important role in promoting physical activity and FMS in educational settings [17, 18]. Classroom teachers and ECEs can incorporate opportunities for engagement in physical activity and the development of FMS into children’s day through active lessons [19], active breaks [20], and active play opportunities [21]. However, whether a classroom teacher or ECE is provided the support and training necessary to improve their capacity to implement physical activity and FMS opportunities may play a crucial role in determining the degree to which these opportunities are implemented in educational settings [22, 23].

The capability motivation opportunity-behaviour (COM-B) model provides a framework to examine the barriers and facilitators to teachers engaging students in physical activity and improving FMS [24, 25]. It positions the interactions between an individual’s capabilities, their motivation, and the opportunities afforded to them by the physical and social environments as key predictors of an individual’s behaviour and behaviour change [24] Capability is defined as an individual’s psychological and physical resources necessary to participate in a behaviour [24]. A commonly cited barrier in this domain is inadequate training, whereas common facilitators in this domain include positive perceptions of capabilities (e.g., self-efficacy) and physical activity knowledge [23, 25] For example, in their review, Nathan et al. [26] found that 89% and 31%, of school teachers reported physical activity-related knowledge and beliefs about capabilities as facilitators for implementing physical activity policies and programs, respectively; the two most commonly referenced facilitators. Motivation is defined as the cognitive processes which encourage behaviour change [24]. Positive attitudes towards promoting physical activity and positive perceptions about the consequences of physical activity are both important facilitators to implement physical activity practices in these settings [23, 25]. Lastly, opportunity encompasses all the factors outside of the individual which may facilitate or hinder behaviour change [24]. A commonly cited barrier among teachers is a lack of availability of resources and space to promote physical activity [23, 25]. Barriers that hinder the promotion of physical activity by teachers and ECEs may also inhibit educators from teaching children FMS [27]. Therefore, interventions that increase teachers’ capability and motivation to facilitate physical activity and FMS and improve opportunities may be an effective strategy to promote more active educational settings for children.

One approach to improving an teachers’ capability and motivation and opportunity is through capacity building. Capacity building is defined by the WHO as “the development of knowledge, skills, commitment, structures, systems and leadership to enable effective health promotion” (p. 341) [28]. Capacity building may take place at the individual or organizational level and includes such things as training and workshops, provision of resources, communities of practice, ongoing technical assistance, and designing and implementing policies to institutionalize health promotion [28–30]. Capacity building can be general and focus on building an individual’s and organization’s capacity to implement any intervention, or capacity building may intervention specific where the focus is on building the motivation and skills necessary to implement a specific intervention [31]. The interactive systems framework for dissemination and implementation positions capacity building at the centre of successfully implementing evidence informed practices and interventions in practice [30]. Furthermore, models of teacher professional growth and development position capacity building at the beginning of a causal chain to improve student outcomes through increase in teachers’ knowledge, attitudes and skills [32], or as part of a complex integrative process or continual of learning and professional growth [33]. Altogether, capacity building is positioned as an important activity to support teacher, and ultimately, student outcomes. There has been some research which has demonstrated the effectiveness of capacity building interventions on improving public health practitioners’ knowledge, perceived capabilities, skills, and behaviours [29]. However, the effect of physical activity capacity building interventions in classroom teachers and ECEs is unclear. Therefore, the aim of this study was to synthesize the effect of capacity building interventions on teachers’ and ECEs’ perceived capabilities to promote physical activity and FMS, their physical activity and FMS related knowledge, and their attitudes towards promoting physical activity and FMS.

## Methods

This systematic review and meta-analysis was conducted in accordance with the Preferred Reporting Items for Systematic Reviews and Meat-Analyses (PRISMA; Appendix A) and was pre-registered in the International Prospective Register for Systematic Reviews database (PROSPERO; CRD42022330335).

### Information sources and search

Primary literature searches were conducted in Medline (via OVID), SPORTDiscus, PsychInfo, Cumulative Index to Nursing and Allied Health Literature (CINAHL), Education Resources Information Center (ERIC), and Scopus, with the final search conducted on May 5th 2022 and included all records from the inception of each database. The search was limited to titles and abstracts written in English. Searches used keywords for capacity building interventions, physical activity, teachers and ECEs, and intervention studies, and were combined using Boolean operators. The full list of search terms can be found in Appendix B. Primary literature searches were supplemented by manual screening of the reference list of included studies, and reverse screening of studies that referenced included studies using Scopus.

### Eligibility criteria

#### Participants

Studies were eligible if they included pre-service (i.e., in-training) or in-service classroom teachers (i.e., teachers without specialized training in physical education) or ECEs in early childhood education, primary/elementary education, or secondary education. Because the focus of this review was on teachers and ECEs who may lack the capacity to implement physical activity and FMS interventions, studies including specialist PE teachers (i.e., teachers specifically trained PE) were excluded. Studies were also excluded if they included other school/childcare personnel who were not teachers or ECEs in their analytic sample.

#### Interventions

All capacity building interventions were eligible. For the purpose of this review, capacity building interventions were defined as interventions that aimed to increase educators’ capacity to increase young people’s engagement in physical activity through the advancement of knowledge and skills, the expansion of support and infrastructure, the development of partnerships with others, or through the development and institutionalization of policies to promote favourable practice.

#### Study designs

Controlled (including randomized and non-randomized), single group pre-post, and cross-sectional case control studies were eligible for inclusion.

#### Outcome measures

Studies were eligible for inclusion if they examined teachers’ or ECEs’ perceived capabilities to increase children’s physical activity and FMS (e.g., self-efficacy, perceived behavioural control, perceived competence), physical activity and FMS related knowledge, or their attitudes towards promoting physical activity and FMS development in young people. Validated and made-for-purpose self-reported scales were included. Both test-based and perception-based knowledge questionnaires were included. Scales that measured outcomes for physical activity and sedentary behaviour simultaneously were included; however, scales that measured outcomes exclusively for screen time and sedentary behaviours were excluded. Scales that measured outcomes for physical activity or FMS and any other health behaviour (e.g., healthy eating) simultaneously were excluded, as it is not possible to differentiate the effect of the interventions on physical activity or FMS and other health behaviours.

### Study selection

Search results were saved in Covidence (Veritas Health Innovation, Melbourne, Australia, https://www.covidence.org), where duplicates were automatically removed. Unique references were then uploaded to ASReview. ASReview is an open-source machine learning program which uses active learning to assist in the review process [34]. The machine learning model was trained with prior knowledge by manually labelling three studies for inclusion and 10 studies for exclusion. The model then ranks the remaining articles in order of their likelihood of inclusion and the reviewer manually labels whether the study presented by ASReview is either relevant or irrelevant. After each selection, the model is retrained. For this review, ASReview’s default active learning model was used (see Van de Schoot et al., 2021 for details). Three independent reviewers each completed the title and abstract screening of the relevance ranked list. Each reviewer kept screening titles and abstracts until the following two criteria were met: (a) 30% of all titles and abstracts were screened, and (b) 500 consecutive titles and abstracts were labelled as irrelevant by the reviewer. Simulation studies have demonstrated that in reviews with greater than 5,000 unique records, 100% of relevant records are found in the first 30% of articles screened using ASReview [34]. All articles that were labelled as relevant by at least two reviewers were retrieved for full text screening. Titles and abstracts of articles that were labelled as relevant by a single author were re-reviewed by the lead author who made the final decision on whether full texts were retrieved. Non-published master and doctoral theses that were identified in the search and met the inclusion criteria were included in the review.

Full text screening was completed for each potentially relevant article in Covidence by two independent reviewers. Where there were discrepancies between reviewers, the lead author reread the full text and made the final decision on whether it was included. After a list of full texts was finalised, each reviewer screened the reference list of included studies to identify any potentially relevant articles not identified in the database search. Additionally, more recently published articles that cited included studies were identified using Scopus. A list of potentially relevant articles was made, and the lead author reviewed the full-text of each article to determine its appropriateness for inclusion in the review.

### Data extraction

A single author extracted data into a standardized, pre-piloted extraction template in Microsoft Excel (Microsoft Corporation, Redmond, WA). Data was extracted for sample characteristics (average age, percent female, type of educator, level of educator), outcome measures (i.e., perceived capability, knowledge, and attitudes), study design and duration, and intervention characteristics (e.g., capacity building components). Capacity building components include training and professional development (i.e., in-person or online courses/lectures/modules), resources and toolkits (i.e., providing support materials or physical resources useful for the implementation of physical activity in the classroom), communities of practice (i.e., developing networks to share knowledge experiences, thoughts, etc. among members), mentorship (i.e., one-on-one support provided by a physical activity/FMS expert), ongoing support (i.e., email, phone, or in-person technical support provided to teachers after the completion of initial training/professional development), and policy (i.e., develop and implement policies which support teachers to implement physical activity). WebPlotDigitizer (Ankit Rohatgi, Pacifica, CA, https://automeris.io/WebPlotDigitizer) was used to extract numerical data from graphs where values of the mean and standard deviations of the outcomes were not presented in the text. WebPlotDigitizer has demonstrated validity and intercoder reliability [35]. All extracted data was cross-checked by another reviewer to ensure accuracy.

### Risk of bias/quality assessment

Risk of bias for randomized controlled trials was assessed using the RoB2 tool [36] and the ROBINS-I tool was used for non-randomized controlled trials [37]. Quality of single group pre-post studies with no control group was assessed using the National Institute for Health’s Quality Assessment Tool for Before-After (Pre-Post) Studies With No Control Group [38]. Risk of bias assessments were conducted by two-independent reviewers. Discrepancies were resolved through a discussion between the reviewers until a consensus was achieved. The overall risk of bias for each study was assessed using procedures outlined in the original tools. For randomized controlled trials, the overall risk of bias was rated as low if it had a low risk of bias in all domains, some concerns if the study raised some concerns in at least one domain, and as high if the study had a high risk of bias in at least on domain or had some concerns in multiple domains [36]. The overall risk of for non-randomized controlled trials was rated as low if it had a low risk of bias in all domains, moderate if it had a low or moderate risk of bias for all domains, serious if it had a serious risk in at least one domain and critical if it had a critical risk in at least one domain [37]. For single group pre-post studies, overall study quality was rated based on the reviewer’s judgment of the risk measurement bias, selection bias, information bias, and confounding [38]. The overall quality of these studies was rated as poor, fair, or good.

### Calculation of effect size

The standardized mean difference (SMD; Hedge’s g) for perceived capabilities, knowledge, and attitudes were calculated for continuous variables using raw score metrics [39]. The SMD for continuous variables reported in controlled trials was calculated as the difference in the mean change from baseline to post-intervention between the intervention and control group, divided by the pooled standard deviation from baseline. For continuous outcomes reported in single group pre-post studies, the SMD was calculated as the mean change from baseline to post-intervention, divided by the standard deviation at baseline.

For dichotomous variables reported in controlled trials, the difference in log odds-ratio comparing baseline to post-intervention for the intervention and control group were calculated. For single group pre-post studies, the log odds-ratio comparing baseline to post-intervention was calculated. Log odds-ratios were converted into SMDs by dividing the log odds-ratios by $\frac{\pi }{\sqrt{3}}$ [40]. All SMDs were adjusted for the upward bias of small samples sizes [41].

Variances in effect sizes were calculated using the equations specified by Borenstein et al. (2009). The variance of log odds-ratios were converted to the variance for SMD by dividing by$\frac{{\pi }^{2}}{3}$. For single sample pre-post studies, the correlation between outcomes between baseline and post-intervention were calculated and used to estimate variance. Where a correlation could not be calculated the median of the correlations for all other studies were used for each outcome which was 0.38 for perceived capability and 0.45 for attitudes. No correlations could be calculated for knowledge so a conservative correlation of 0.40 was used to calculate the variance of effect sizes.

### Statistical analysis

Statistical analyses were completed in RStudio v. 1.3 (RStudio Team, Boston, MA), using the robumeta [43], clubSandwhich [44], metaphor [45], and PublicationBias [46] packages. Random-effects meta-analyses were used to estimate the pooled effect sizes for each outcome. To account for non-independent effect sizes within studies for perceived capabilities, a robust-variance estimator (RVE; Hedges et al., 2010; Tanner-Smith & Tipton, 2014) was used to calculate the pooled effect size. RVE provides non-biased estimates of effect sizes and their variances, even when the exact covariance structure is unknown [47, 48]. The analysis used approximately inverse variance averaged weights assuming a correlation of 0.8 between effect sizes within studies. A small sample size adjustment was applied to the estimation [49]. Between study variance is estimated using a method of moments estimator [48]. For knowledge and attitudes, the average number of non-independent effect sizes within studies was small (i.e., < 1.5). Therefore, effect sizes within studies were combined before analysis so that each study only had an individual effect size, and a pooled effect size was estimated using the generic inverse variance method. Between study variance was estimated using the restricted maximum likelihood method [50]. Between study heterogeneity was assessed using I^2^ [51] and an approximate 95% prediction interval [52]. I^2^ presents the proportion of between study variance of the total variance and the 95% prediction interval provides a range of likely effects in similar future studies. A sensitivity analysis was conducted for each outcome excluding randomized controlled trials which were rated as a high risk of bias, non-randomized controlled trials rated as having a serious or critical risk of bias and single group pre-post studies rated as poor quality.

Subgroup analysis was conducted for perceived capabilities. Planned subgroup analysis was conducted for type of teacher (early childhood educator, elementary/primary teacher) and a planned meta-regression was run to estimate the effect of the length of the study. Based on available data, a subgroup analysis was also conducted to examine differences between study designs (control group, single group), teacher level (pre-service, in-service), and theoretical basis of the intervention (theoretically informed, atheoretical), and meta-regression was conducted to determine if the number of capacity building components was related to changes in perceived capabilities. As suggested by Tipton and Pustejovsky (2015) the approximate Hotelling’s ${T}_{Z}^{2}$ test was used to test moderators for perceived capabilities. Given the limited number of studies and unequal distribution of effect sizes between subgroups, subgroup analysis was not estimated for knowledge or attitudes.

Publication bias was assessed through the examination of funnel plot asymmetry. Funnel plot asymmetry was estimated using Egger’s regression [54]. To avoid an artificial correlation between an SMD and its standard error, the SMD was removed from the equation when calculating the standard error for Egger’s regression [55]. Additionally, to determine how sensitive the result of the meta-analyses were to publication bias, the methods described by Mathur and Vanderweele [46] were used to calculate a fail-safe n to determine the number of non-affirmative studies that would need to be published to lead to null results. The unpublished non-affirmative results are assumed to be comparable to published non-affirmative results. Tests of publication bias were only conducted for meta-analyses with at least 10 independent effect sizes [51].

## Results

### Study selection

**Fig. 1:**
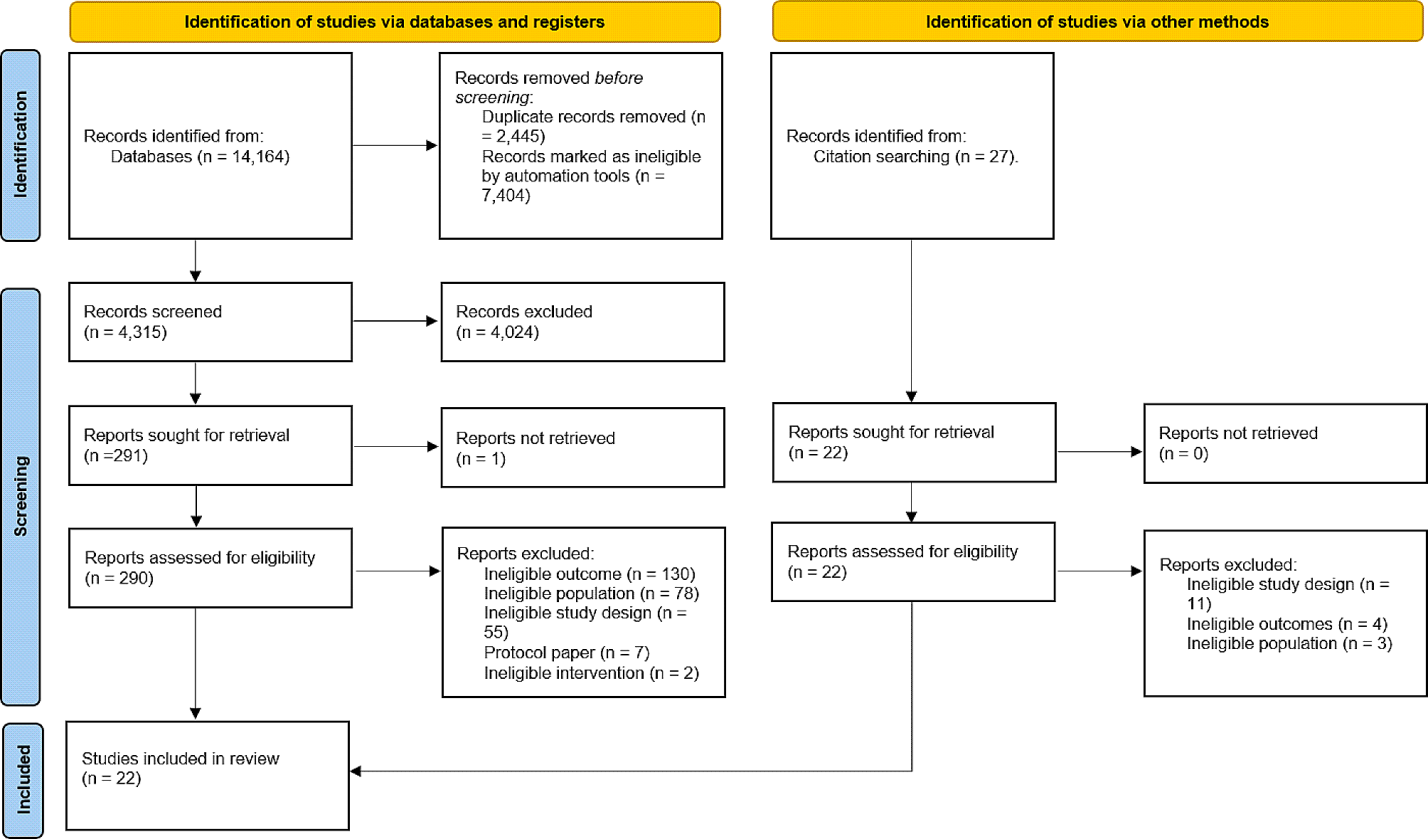
PRISMA flowchart of study screening and selection

After removing duplicates, the literature search yielded 11,720 potentially relevant articles. Of these, 4,315 titles and abstracts were screened manually, and the reaming 7,404 were ranked below the threshold for manual screening. Each reviewer screened a slightly different pool of titles and abstracts based on their unique machine learning algorithm. Overall, 795 titles and abstracts were screened by a single reviewer, 804 titles and abstracts were screened by two reviewers, and 2,716 titles and abstracts were screened by all three reviewers. A total of 292 full text articles were sought for retrieval. One full text could not be retrieved, so 291 full texts were reviewed. Of these, 273 were excluded and 18 relevant articles met all inclusion criteria. Four additional articles were identified by checking and reverse checking reference lists. In total 22 articles, reporting on 25 unique samples were included (Fig. 1).

### Study characteristics

Characteristics for the included studies are displayed in Appendix C. Studies were conducted in the United States (k = 7), Australia (k = 6), Canada (k = 5), Turkey (k = 3) and Ireland (k = 1). Of the included articles, 14 employed a single group pre-post design [56–69], four were randomized controlled trials [70–73], and three were non-randomized controlled trials [74–76]. One study was a randomized control trial, however, only reported on the outcomes of interest in the intervention group [77]. Almost all studies reported on ECEs (k = 10) or elementary/middle school teachers (k = 10). Additionally, one study reported on both ECEs and elementary teachers and one study did not report the type of school. No studies reported on secondary school teachers. Overall, 13 studies reported on in-service teachers, eight reported on pre-service teachers, and one reported on both pre-service and in-service teachers. The proportion of study populations that was female ranged between 40 and 100% (median = 94.8%). The sample size of included studies ranged from five to 308 (median = 56) and studies ranged in length from 2 weeks to 9 months. By far and away, the capacity building interventions described in the literature focused on individual level capacity building. The most common capacity building components reported in studies included training/professional development (19 studies), resources and toolkits (12 studies), communities of practice (10 studies), mentorships (seven studies), and ongoing support (four studies). Twenty of the 25 interventions included multiple capacity building strategies (median = 2.00). Overall, 11 studies reported the model that was used to inform their intervention. Some studies reported using multiple models. Ten of these reported on behaviour change models including the Social Cognitive Theory (k = 8), ecological models (k = 5), Behavioural Choice Theory (k = 2), and Self-Determination Theory (k = 1). A single study reported using a Cooperative framework to teacher professional development.

### Risk of bias/study quality

The results from the assessment of risk of bias and study quality are displayed in Appendix C. For randomized controlled trials, three studies were rated as having some concerns overall while two were rated as having a high risk of bias, mainly owing to risk of bias from the randomization process and selective reporting. The two non-randomized controlled trials were both rated as having a high risk of bias overall, owing to risk of confounding, risk in the selection of participants, and risk due to the measurement of the outcome. Of the single group pre-post studies, four were rated as good quality, six were rated as fair quality, and three were rated as poor quality. Studies were rated as poor quality for not having a clear inclusion and exclusion criteria, not performing appropriate statistical tests, and having small sample sizes.

### Meta-analysis

#### Perceived capability

**Fig. 2:**
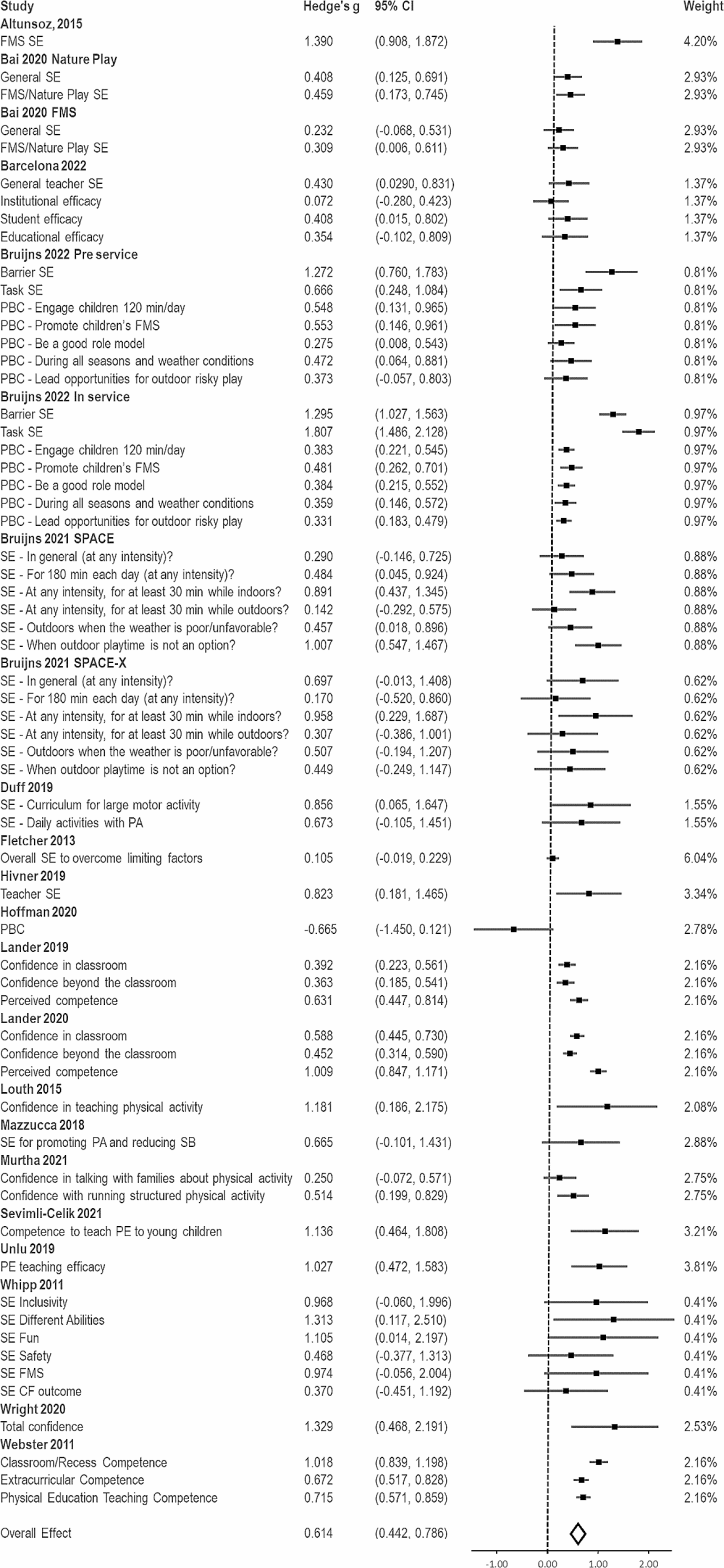
Forest plot of effect sizes from the robust variance meta-analysis for perceived capability

A total of 19 studies reported on 22 unique samples and 62 effect sizes, with a combined sample size of 1,849, were included in the meta-analysis for perceived capability. Results demonstrated a medium pooled effect (g = 0.614, 95% CI = 0.442, 0.786; Fig. 2); however, there was substantial between-study heterogeneity (I^2^ = 85.60%) and the 95% prediction interval was − 0.179 to 1.407. The results from the Egger`s regression was non-significant (*p* = .365), indicating that there was not significant funnel plot asymmetry (Appendix D). Results from the fail-safe n indicated that no number of non-affirmative results could sufficiently attenuate the results so they become null.

Results from the sensitivity analysis excluding studies with a high risk of bias or low study quality demonstrated a somewhat attenuated pooled effect (k = 14, g = 0.486, 95% CI = 0.298, 0.673), with similar between study heterogeneity (I^2^ = 89.13%, 95% prediction interval = -0.331, 1.303).

Results from the subgroup analysis and meta-regression show that the estimated effect size did not significantly differ across any of the subgroups tested and was not related to the length of the study (Table 1).

**Table 1:** Results from the subgroup analysis for perceived capabilities

	j	df	Hedge’s g	95% CI	Moderator *p*-value
Design					0.777
Independent group	18	5.76	0.665	0.103, 1.227	
Single group pre-post	44	12.70	0.595	0.423, 0.768	
Type of teacher^a^					0.879
Early Childhood Education	38	10.40	0.572	0.303, 0.841	
Elementary	20	6.02	0.601	0.263, 0.938	
Teacher level					0.303
Pre-service	20	6.73	0.722	0.381, 1.062	
In-service	42	11.80	0.537	0.321, 0.753	
Intervention theory					0.361
Theoretically informed	30	8.35	0.533	0.278, 0.788	
Atheoretical	32	10.01	0.691	0.411, 0.971	
Study length (weeks)^b^	62	4.24	-0.009	-0.028, 0.011	0.299
Number of capacity building components	62	8.39	-0.069	-0.281, 0.143	0.477

*Note* j = number of effect sizes, df = degrees of freedom, CI = confidence interval

^a^ Number of effect sizes do not add up to 62 as some studies did not report on the type of teacher or included multiple types of teachers

^b^ Intervention length was treated as a continuous variable in the meta-regression

#### Knowledge

**Fig. 3:**
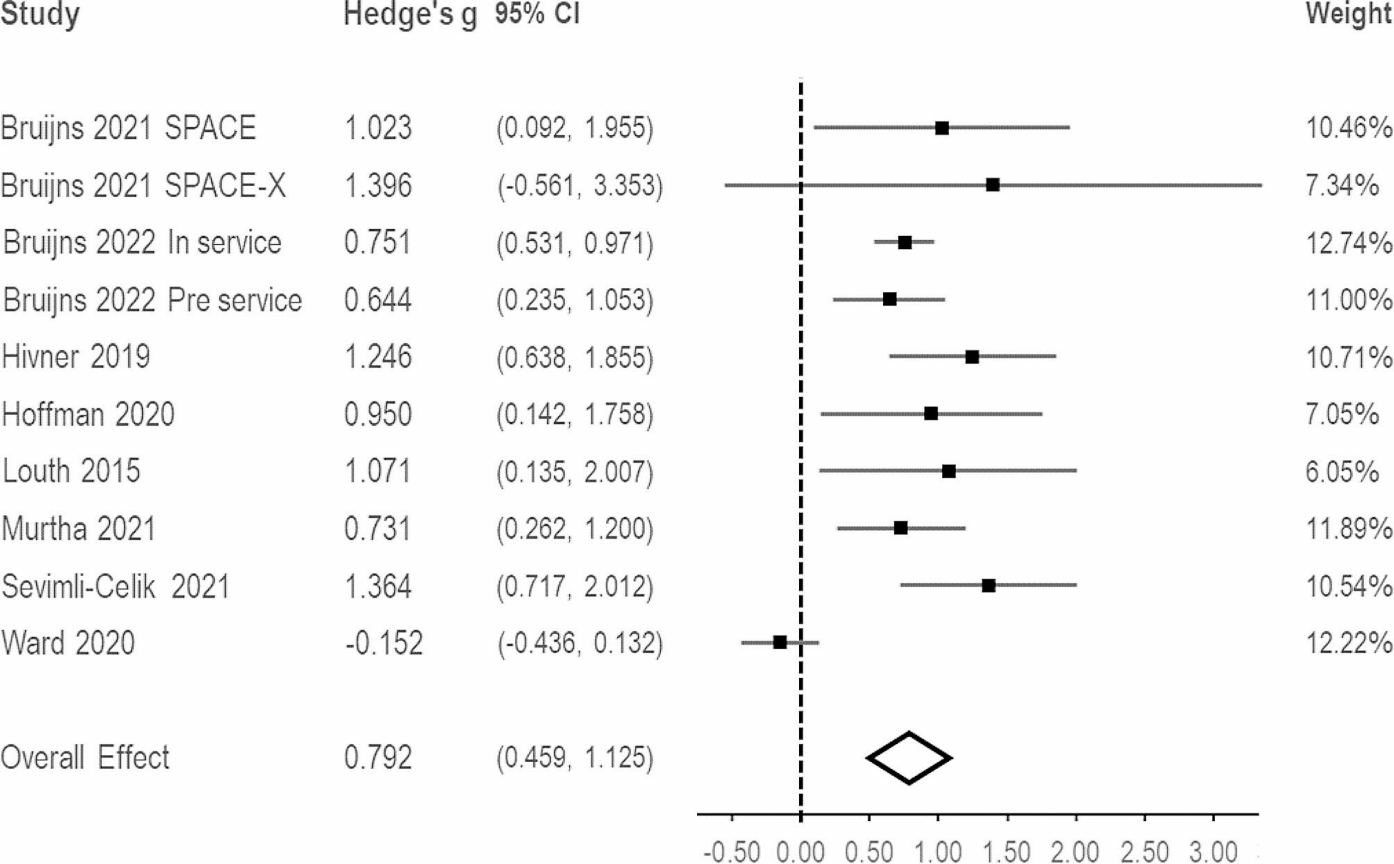
Forest plot for effect sizes from the random effects meta-analysis for physical activity-related knowledge

A total of eight studies reporting on 10 independent effect sizes and 642 participants were included in the meta-analysis for knowledge. There was a large pooled effect for this outcome (g = 0.792, 95% CI = 0.459, 1.125; Fig. 3) and there was substantial between-study heterogeneity (I^2^ = 75.52%) with a 95% prediction interval of -0.239 to 1.824. The results from the Egger`s regression were non-significant (*p* = .067) indicating that there was not significant funnel plot asymmetry (Appendix D). Results from the fail-safe n indicated 22 non-affirmative results could sufficiently attenuate the results so they become null.

Results from the sensitivity analysis excluding studies with a high risk of bias or low study quality demonstrated a somewhat attenuated pooled effect (k = 6, g = 0.600, 95% CI = 0.166, 1.036), with increased between study heterogeneity (I^2^ = 82.70%, 95% prediction interval = -0.648, 1.743).

#### Attitudes

**Fig. 4:**
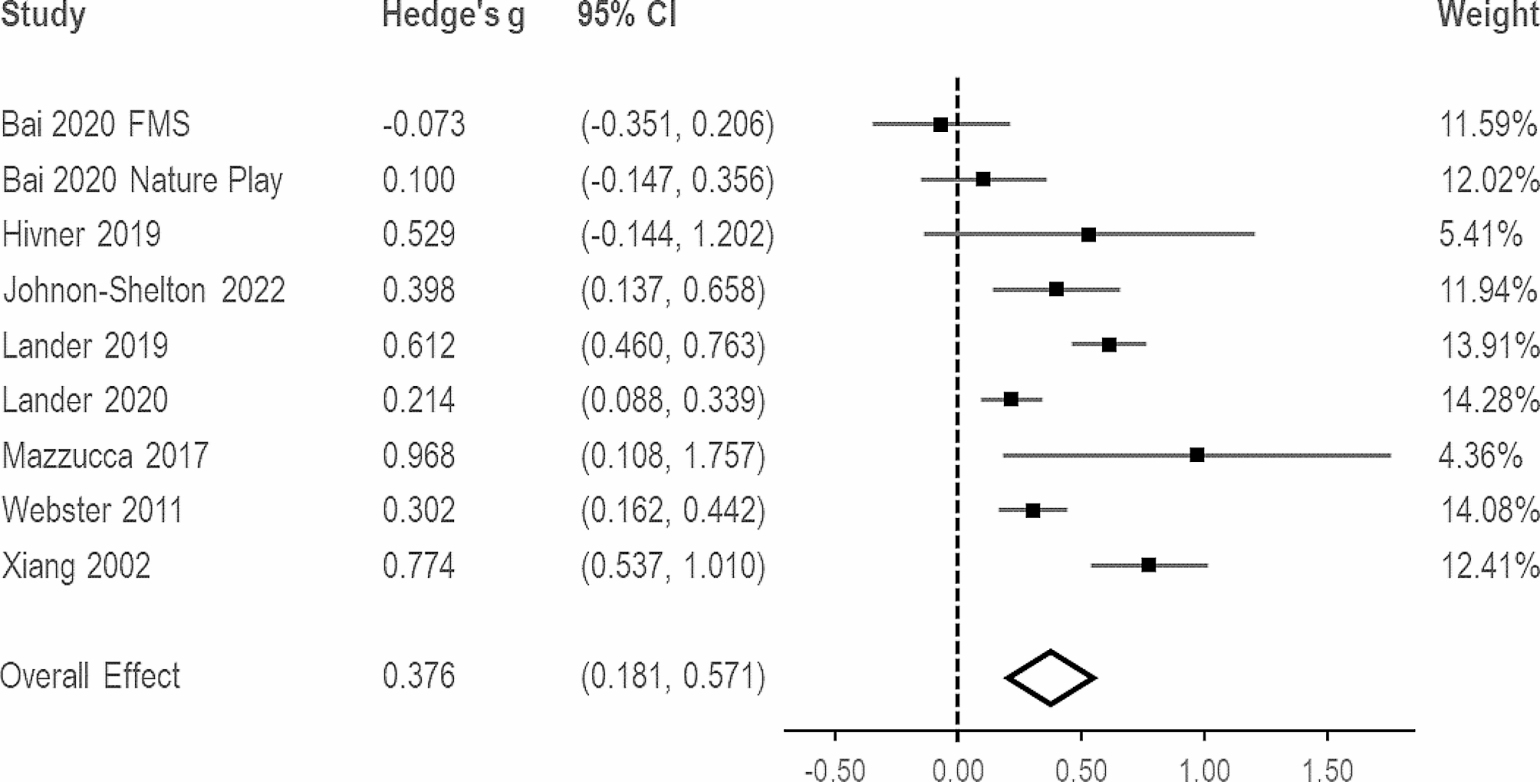
Forest plot for effect sizes from the random effects meta-analysis for attitudes towards promoting physical activity

A total of eight studies reporting on nine independent samples and including 1,112 participants were included in the meta-analysis for attitudes. The pooled effect for this outcome measure was modest (g = 0.376, 95% CI = 0.181, 0.571; Fig. 4). There was substantial between-study heterogeneity (I^2^ = 84.98%) and the 95% prediction interval was − 0.256 to 1.008. The funnel plot of effect sizes can be seen in Appendix D.

Results from the sensitivity analysis excluding studies with a high risk of bias or low study quality demonstrated an almost identical pooled effect (k = 8, g = 0.372, 95% CI = 0.164, 0.581), and between study heterogeneity (I^2^ = 85.70%, 95% prediction interval = -0.312, 1.056).

## Discussion

Classroom teachers and ECEs play an important role in ensuring children engage in sufficient levels of physical activity and have the movement skills necessary to engage in lifelong physical activity [17, 18]. Teachers’ capabilities, attitudes, and the opportunities afforded by the environments in which they work may all simultaneously and synergistically influence their behaviours related to promoting physical activity and FMS [24, 25]. Capacity building interventions, either at the individual or school level, have the potential to increase teachers’ and ECE’s capacity to incorporate physical activity and FMS into the curriculum through increasing their knowledge, perceptions about their capabilities (e.g., self-efficacy), and improving attitudes towards physical activity and FMS [22, 23, 78]. Therefore, this meta-analysis evaluated the effect of capacity building interventions on teachers’ and ECEs’ capacity related to physical activity and FMS. Results suggest that capacity building interventions were effective at increasing both teachers’ and ECEs’ perceived capabilities (g = 0.614), knowledge (g = 0.792), and attitudes (g = 0.376) related to physical activity and FMS. Based on models of teacher growth [32, 33], these improvements in teacher outcomes are likely to positively influence student outcomes through changes in teachers’ practices. These findings speak to the value of capacity building interventions to foster more active educational settings.

Generally speaking, the capacity building interventions identified in this review focused on capacity building strategies at the individual level. Almost all studies included in the current review provided teacher training or professional development as a capacity building component of the intervention. The results from this meta-analysis demonstrate that providing teacher training or professional development opportunities are likely to increase teachers’ and ECEs’ capacity to implement physical activity opportunities into the curriculum, and highlight the value of giving classroom teachers and ECEs physical activity and FMS specific training. However, most interventions included multiple capacity building components, including training in combination with continued support, communities of practice, resources and toolkits, or mentorship. Previous research has demonstrated that taking a multi-strategy approach towards teacher capacity building can lead to increase implementation of physical activity opportunities in schools [79, 80], and may be more effective than providing teacher training alone [81]. As such, it is promising to see that most capacity building interventions are taking a multi-strategy approach. Although most studies took a multi-strategy approach to capacity building, very few studies utilised strategies to build capacity at both the individual and organizational level. A range of factors across various levels of influence, including organizational capacity (e.g., support from leadership) and community level (e.g., funding) have the potential to facilitate or inhibit implementation of physical activity programs and policies in school settings [22, 23]. Indeed, the competency of individuals itself may not be sufficient to achieve health promotion goals, and support from the organizations that they work within is equally important [28]. Therefore, more research is needed to determine if incorporating strategies to increase teachers’ and ECEs’ capacity alongside strategies aimed at improving organizational capacity, such as policy implementation, environmental modifications, support from leadership, and increased funding, are more effective at increasing teachers’ and ECEs’ knowledge, competence, and attitudes towards implementing physical activity and FMS opportunities in the curriculum.

An important finding from this meta-analysis is that capacity building interventions were effective at increasing perceived capabilities in both pre-service and in-service teachers. The limited number of studies precluded a formal subgroup analysis comparing the effects of capacity building interventions on knowledge and attitudes in pre-service and in-service teachers and ECEs. Nevertheless, results from individual studies included in the meta-analysis indicate that capacity building interventions can increase pre-service teachers’ and ECEs’ physical activity-related knowledge [63, 69] and attitudes [59, 60, 82]. Therefore, in addition to improving in-service teachers’ and ECEs’ physical activity-related practices, capacity building interventions have the potential to increase pre-service teachers’ and ECEs’ capacity to promote physical activity when they enter the profession. However, teachers and ECEs may not be given the opportunity to engage in formalised training in physical activity and FMS [14, 15]. Nevertheless, embedding physical activity content into the pre-service training of teachers is perceived as a feasible, acceptable, and even necessary by a range of stakeholders including university lecturers, pre-service teachers, and school administrators to equip teachers with the pedagogical skills necessary to promote physical activity in the classroom [59, 60]. Providing pre-service teachers and ECEs with the requisite skills, knowledge, and attitudes to promote physical activity in their classrooms could have far-reaching positive impacts on children’s physical activity.

Although the results from this meta-analysis demonstrated the effectiveness of capacity building interventions on teacher outcomes, an important consideration to make is that reach of the studies included in this systematic review and meta-analysis were relatively limited, with the median sample size of included studies being 56 teachers or ECEs. An important area of future research is to consider the scalability of capacity building interventions. Large-scale capacity building interventions may be a necessary component of the implementation of district, state, or national regulations relating to physical activity, which may have inadequate adherence [83, 84] and limited effect at increasing children’s engagement in physical activity when implemented alone [85]. Recently, an evaluation of a large scale capacity building initiative, implemented alongside the introduction of a state legislated active play policy in childcares [86], was conducted with over 1,800 childcare staff (including ECEs, and other support staff) [87]. The capacity building intervention, which included training, toolkits, technical support and communities of practice, used a train-the-trainer approach, where regional trainers were given the skills necessary to deliver the capacity building intervention in their municipality, and was successful at increasing childcare staff’s capacity to increase children’s physical activity [87]. Interestingly, the effect of the capacity building intervention was largely consistent across modalities including in-person, live online workshops, and e-learning modules [87]. However, findings relating to the effectiveness of virtual capacity building interventions from the current review were mixed, with some studies showing that virtual interventions may be effective [69], while others had mixed or null findings [71, 73]. Given the potential reach and cost effectiveness of delivering capacity building interventions online, further research is warranted to examine the effectiveness of online interventions.

There are several research gaps that can be identified from the results of the current systematic review and meta-analysis that warrant further attention. No studies were identified that examined the effect of capacity building interventions on secondary school classroom teachers’ perceived capabilities, knowledge, or attitudes. This may be because secondary schools are more likely to have specialised PE teacher and formalized PE curriculums than primary schools [12]. However, given that classroom teacher led physically active lessons may be an effective strategy to improve adolescents’ physical activity, fitness, and on-task behaviour in secondary school students [88, 89], it may be important to consider how capacity building interventions affect secondary school classroom teachers. Second, the vast majority of studies were conducted in high income countries. It is important to determine the effectiveness of capacity building interventions in more diverse contexts and examine if teachers from low-to-middle income countries would benefits from unique or additional capacity building components. Additionally, while validated tools exist to measure the outcomes included in this review [90–93], most studies included in this review employed scales that were not validated. Future research should use validated measures of teachers’ and ECEs’ perceived competence and attitudes relating to physical activity and FMS whenever possible. Further, while some studies included measures which simultaneously referred to physical activity and screen time/sedentary behaviours, future research may consider examining these outcomes related to these behaviours independent of physical activity.

### Strengths and limitations

This review is the first meta-analysis to examine the effect of capacity building interventions on teachers’ and ECEs’ perceived capabilities, knowledge, and attitudes related to physical activity and FMS. This review used a machine learning classifier to assist in title and abstract screening. This is advantageous as it allows for the expansion of search terms; and therefore, the identification of a greater number of potentially relevant articles, without becoming overwhelming for reviewers. Additionally, the extremely imbalanced nature of data found in database searches (i.e., significantly more irrelevant than relevant titles and abstracts) means that traditional reviews may be error prone [94]. By displaying the most relevant articles first, using the machine learning algorithm may reduce the error rate when identifying relevant articles from titles and abstracts. Additionally, a thorough approach to reviewing articles cited in and cited by all included studies was implemented to ensure that relevant articles missed in the original search were included in the review. There are also some limitations to the current review that should be taken into consideration. First, this review was limited to articles written in English, potentially omitting relevant studies written in other languages, and introducing an English language bias [95]. Second, the database search was conducted in May 2022, and was already somewhat outdated by the time this review was published. Third, the review included both controlled trials and single group pre-post studies. There are several compelling reasons to exclude single group pre-post studies from a meta-analysis [96]. However, given that the extant literature consists of a majority single group pre-post studies, it was not viable to exclude them from the current meta-analysis. Raw score metrics as described by Morris and DeShon [39] were used to calculate all effect sizes, so they were comparable across study designs. Additionally, the results from the subgroup analysis indicated that study design did not significantly moderate the estimated effect size for perceived capabilities, increasing confidence that it was appropriate to combine study designs. Lastly, many of the included studies were rated as having a high risk of bias or as poor quality. This may have implications for the reliability and validity of the reported results and more high-quality studies are needed to confirm the results of this meta-analysis.

## Conclusion

Findings from this meta-analysis suggest that capacity building interventions are effective at increasing teachers’ and ECEs’ perceived competence, knowledge and attitudes related to physical activity and FMS. The most common capacity building intervention component implemented in the identified studies was teacher training/professional development, highlighting the value of providing training to increase teachers’ and ECEs’ capacity to increase physical activity and improve FMS in their students. Training should be provided to teachers and ECEs at both the in-service and pre-service level. Capacity building may also be an important component to the implementation of physical activity policies and mandates to ensure adherence.

### Electronic supplementary material

Below is the link to the electronic supplementary material.

Supplementary Material 1

Supplementary Material 2

Supplementary Material 3

Supplementary Material 4

Supplementary Material 5

## Data Availability

Contact Matthew Bourke to request access to the extracted data and analysis code.

## Abbreviations

CINAHL: Cumulative Index to Nursing and Allied Health Literature
ECE: Early Childhood Educator
ERIC: Education Resources Information Center
FMS: Fundamental Movement Skills
PE: Physical Education
PRISMA: Preferred reporting Items for Systematic Reviews and Meta-Analyses; PROSPERO: International Prospective Register for Systematic Reviews
SMD: Standardized Mean Difference
RVE: Robust Variance Estimator
