# Supplementary material for: Effect of capacity building interventions on classroom teacher and early childhood educator perceived capabilities, knowledge, and attitudes relating to physical activity and fundamental movement skills: a systematic review and meta-analysis
Source: BMC Public Health. 2024 May 27;24:1409. doi: 10.1186/s12889-024-18907-x (PMC11129429; doi:10.1186/s12889-024-18907-x)
Supplement: Supplementary file 1 — Supplementary Material 1 [file 12889_2024_18907_MOESM1_ESM.pdf]

Appendix B – Search terms

(“capacity building” OR “capacity development” OR “professional development” OR “professional learning” OR “staff development” OR “continual development” OR “continual improvement” OR “technical support” OR “support network” OR toolkit OR “community of practice” OR trained OR training) AND (“physical* activit*” OR exercis* OR “gross motor” OR “fundamental movement skills” OR “physical education” OR “active* break*” OR “active play” OR movement OR mvpa) AND (“early childhood” OR “child?care*” OR “pre?school*” OR “primary school*” OR “elementary school*” OR “secondary college*” OR “secondary school*” OR “high?school” OR teacher* OR educator*”) AND ("randomized controlled trial" OR "controlled clinical trial" OR "pragmatic clinical trial" OR "multicenter study" OR "controlled before-after study" OR randomis* OR randomiz* OR randomly OR trial OR multicenter OR multi center OR multicentre OR "multi centre" OR intervention? OR effect? OR impact? OR controlled OR "control group?" OR "before adj5 after" OR "pre adj5 post" OR pretest OR "pre test" OR posttest OR "post test" OR quasiexperiment* OR "quasi experiment*" OR evaluat* OR "time series" OR "time point?" OR "repeated measur*" OR pilot OR “case control”)
