# Supplementary material for: Effect of capacity building interventions on classroom teacher and early childhood educator perceived capabilities, knowledge, and attitudes relating to physical activity and fundamental movement skills: a systematic review and meta-analysis
Source: BMC Public Health. 2024 May 27;24:1409. doi: 10.1186/s12889-024-18907-x (PMC11129429; doi:10.1186/s12889-024-18907-x)
Supplement: Supplementary file 2 — Supplementary Material 2 [file 12889_2024_18907_MOESM2_ESM.pdf]

Table C1 – Overview of included studies

Author	Country	Sample^	Theoretical framework	Study design, setting and duration	Capacity building strategies	Outcomes Measured
Altunsöz, 2015	Turkey	Pre-service ECEs<br>N = 83<br>100% female<br>M age = 21.4 yrs	Not reported	Non-randomized control trial<br>In person (university campus)<br>14 weeks	***Training/professional development***<br>A 14 week PE and games course consisting of theoretical and practical sessions covering flexibility, safety procedures, developmental PE, movement concepts, movement skill acquisition, FMS, teaching PE, games, health related fitness, and physical activity for young children<br>***Communities of practise***<br>Participants engaged in group discussions, and gave group presentations to their peers.<br>***Mentor***<br>A trained instructor watched participants implement lesson plans and gave feedback on their teaching practices for FMS.	<u>Perceived capabilities</u><br>Teaching Fundamental Motor Skill Self-efficacy Questionnaire [1]
Bai, 2020	Australia	ECEs<br><u>Nature play group</u><br>N = 63<br>100% Female<br>M age = 37.0 yrs<br><br><u>FMS group</u><br>N = 53<br>100% Female<br>M age = 34.15 yrs	Not reported	Single group pre-post (2 groups)<br>In-person (long day care centres)<br>3 months	**<u>Nature play group</u>**<br>***Training/professional development***<br>A two-hour professional development workshop was held with all educators from the day care centres personalized to ECEs' needs. Additionally, a one-hour incursion with a group of children and educators to demonstrate how natural materials can be used to support children's play and learning.<br><br>**<u>FMS group</u>**<br>***Training/professional development***<br>Aimed to provide ECEs with an understanding of the processes of motor development; specifically the development and training of FMS and motor proficiency, and how to engage children in more physical activity whilst at ECE centres.<br>***Mentor***<br>An FMS professional shadowed ECEs to observe educator practices and provide feedback.	<u>Perceived capabilities</u><br>Self-efficacy to engage children to be active in general<br>Self-efficacy to engage children to be active in FMS/nature-based play activities<br>Perceived behavioural control over support for children's FMS/Nature play<br><u>Attitudes</u><br>Importance of physical activity to educators
Barcelona, 2022	United States	Elementary teachers<br>N = 52	Whole school, whole community, whole	Single group pre-post<br>In-person (schools)	***Training/professional development***<br>Over the course of the classroom teachers were provided 2 professional development sessions specific to classroom PA. Sessions	<u>Perceived capabilities</u>

Author	Country	Sample^	Theoretical framework	Study design, setting and duration	Capacity building strategies	Outcomes Measured
		Elementary 52%<br>Middle 12%<br>K-8 5.3%<br>66% Female<br>M age = 39 yrs	child (WSCC) framework.	9 months	introduced research that supported the importance and positive outcomes of routine classroom PA.<br>***Resources***<br>Teachers were introduced to the activity platform GoNoodle, as well as an overview of best practices and strategies for utilizing classroom PA.<br>***Communities of practice***<br>Teachers were encouraged to work in collaboration with other teachers, sharing strategies, and best practices that facilitated implementation of classroom physical activity break in their own classrooms.	Teacher Efficacy Toward Providing Physical Activity in the Classroom Scale [2]
Bruijns, 2022	Canada	<u>Pre-service ECE</u><br>N = 32<br>93.8% Female<br>M age = 26.7 yrs<br><u>In-service ECE</u><br>N = 121<br>99.2% Female<br>M age = 37.1 yrs	Social Cognitive Theory	Single group pre-post<br>Online<br>2 weeks	***Training/professional development***<br>Participants completed a 5-hour e-Learning course in physical activity and sedentary behaviour. The course included introductory content on physical activity and sedentary behaviour in early childhood; the influence of the childcare environment on children's movement behaviours, and outdoor and risky play; practical strategies to promote physical activity and minimize sedentary time among children in childcare.<br>***Resources***<br>Participants were granted access to online resources and a video library with appropriate physical activities.	<u>Perceived capabilities</u><br>ECE Confidence in Outdoor Movement, Physical Activity, Sedentary and Screen Behaviours questionnaire [3]<br>ECE Movement Behavioural Intention and Perceived Control questionnaire [4]<br><u>Knowledge</u><br>Teachers knowledge guidelines, definitions, appropriate behaviours, and facts
Bruijns, 2021	Canada	<u>SPACE ECE's</u><br>N = 83<br>95.9% Female<br>M age = 37.23 yrs<br><u>SPACE-X ECE's</u><br>N = 31<br>100% Female<br>M age = 35.1 yrs	Not reported	Cluster RCT<br>In-person (childcare centres)<br>8 weeks	<u>SPACE</u><br>***Training/professional development***<br>One 4-h training session covering topics including physical activity and sedentary behaviour guidelines for young children, how to facilitate physical activity in childcare, and overcoming barriers to PA.<br>***Policy***<br>Asked childcare centres to modify their play schedule to four daily 30-min outdoor play sessions.<br>***Resources***<br>Provided childcare centres with portable play equipment.	<u>Perceived capabilities</u><br>Self-efficacy to engage pre-schoolers in PA<br><u>Knowledge</u><br>Knowledge of physical activity guidelines

Author	Country	Sample^	Theoretical framework	Study design, setting and duration	Capacity building strategies	Outcomes Measured
					SPACE-X<br>***Policy***<br>Asked childcare centres to modify their play schedule to four daily 30-min outdoor play sessions.	
Duff, 2019	Ireland	ECEs<br>N = 27<br>M age = NR<br>% Female NR	Not reported	RCT<br>In-person (preschools)<br>6 weeks	***Training/professional development***<br>ECEs attended two, two-hour training sessions. The program focused on training educators to integrate physical activity opportunities throughout the preschool day, and to help children improve their performance of FMS<br>***Resources***<br>Teachers were also provided a resource pack with activity cards and posters for FMS and physical activity.	Perceived capabilities<br>Confidence about Activity and Nutrition Teach Questionnaire [5] (Curriculum for large motor activity and Daily activities with physical activity subscales)
Fletcher, 2013	Canada	Pre-service elementary teachers<br>N = 308<br>80.5% Female<br>M age = NR	Not reported	Single group pre-post<br>In-person (university campus)<br>4 weeks	***Training/professional development***<br>Pre-service teachers completed a 12-hour PE methods course delivered by a specialist PE teacher educator. Content included, an introduction to PE curriculum, low organisation games, fitness activities, organised games, and movement experiences.<br>***Community of practice***<br>Pre-service teachers researched a website that could be used as a resource to develop PE lessons, and presented their findings to their peers.	Perceived Capabilities<br>Self-efficacy to overcome barriers to teaching PE
Hivner, 2019	United States	Elementary school teachers<br>N = 41<br>M age = NR<br>% Female NR	Social Cognitive Theory	RCT (analysed as single group)<br>Online & in-person (schools)<br>12 weeks	***Training/professional development***<br>Training was consisted of either one online or one in-person session which focused on topics based in Social Cognitive Theory constructs, including the benefits of classroom physical activity examples and guidelines of implementing activity sessions, solutions to common barriers and the study protocol.<br>***Resources***<br>Participants in both groups received resources to help them plan activity sessions.	Perceived Capabilities<br>Teacher's confidence in their ability to successfully implement classroom physical activity breaks<br>Attitudes<br>Teacher's belief that implementing classroom physical activity will produce positive outcomes<br>Knowledge<br>Teacher's understanding of the importance and appropriate

Author	Country	Sample^	Theoretical framework	Study design, setting and duration	Capacity building strategies	Outcomes Measured
					**Continued support**<br>Participants in the in-person training group also received monthly telephone calls and emails to assist them in implementing classroom activity breaks.<br>**Communities of practice**<br>Participants in the in-person group participated in-group discussions of how to overcome barriers.	methods to incorporate physical activity into the classroom
Hoffman, 2020	United States	ECEs<br>N = 23<br>100% Female<br>M age = 43.6	Social Cognitive Theory	RCT<br>Online & in-person (schools)<br>4 weeks	***Training/professional development***<br>Online training included information about importance of PA, guidelines, role of ECEs in promoting PA, and strategies to increase PA.<br>**Resources**<br>Participants were granted access to an online video library that included examples of structured games, and there was a corresponding game sheet for each game.<br>***Mentor***<br>There was supervisor support that includes observation, feedback, and recognition.	<u>Perceived Capabilities</u><br>Perceived behavioural control towards physical activity promotion<br><u>Knowledge</u><br>Knowledge relating to intervention content
Johnson-Shelton, 2022	United States	Elementary school teachers<br>N = 139<br>80% Female<br>M age = NR	Social Cognitive Theory	Single group pre-post<br>In-person (schools)<br>8 Weeks	***Mentor***<br>During the program, classroom teachers collaborate with professional and community-based physical activity trainers to plan one weekly lesson on a selected activity.<br>***Resources***<br>Teachers are provided with lesson plans which are easy for teachers to use.	<u>Attitudes</u><br>Teacher attitudes toward teaching PE (teacher encouragement and enthusiasm)
Lander, 2019	Australia	Pre-service elementary teachers<br>N = 218<br>76% female<br>Age range 17-47 yrs	Social Cognitive Theory, Behavioural Choice Theory, and Ecological Systems Theory	Single group pre-post<br>In-person (university campus)<br>12 weeks	***Training/professional development***<br>Lectures and seminars; providing the theoretical underpinnings of the importance of PA. Intervention content related to classroom-based physical activity /active teaching, active environments, and active families and based on the social cognitive theory, behavioural choice theory and ecological systems theory.	<u>Perceived capabilities</u><br>Confidence to integrate specific strategies within and beyond the classroom, to increase physical activity and decrease sitting time across the school day—as future teachers

Author	Country	Sample^	Theoretical framework	Study design, setting and duration	Capacity building strategies	Outcomes Measured
					Communities of practice<br>Facilitated peer micro teaching opportunities, whereby the pre-service teachers practice active teaching strategies and receive peer and lecturer feedback.<br>**Resources**<br>Participants were provided with comprehensive lesson plan resources on active teaching, active breaks and active homework.	Perceived competence to effectively integrate specific active teaching strategies across the school day—as future teachers<br>Attitudes<br>Feelings about the impact of increasing activity and breaking sitting time on student outcomes (i.e. on task time, interest, academic outcomes)
Lander, 2020	Australia	Pre-service elementary teachers<br>N = 274<br>Majority aged 17-21<br>76% female	Social Cognitive Theory, Behavioural Choice Theory, and Ecological Systems Theory	Single group pre-post<br>In-person (university campus)<br>12 weeks	***Training/professional development***<br>Lectures and seminars; providing the theoretical underpinnings of the importance of PA. Intervention content related to classroom-based physical activity /active teaching, active environments, and active families and based on the social cognitive theory, behavioural choice theory and ecological systems theory.<br>***Communities of practice***<br>Facilitated peer micro teaching opportunities, whereby the pre-service teachers practice active teaching strategies and receive peer and lecturer feedback.<br>**Resources**<br>Participants were provided with comprehensive lesson plan resources on active teaching, active breaks and active homework.	Perceived capabilities<br>Confidence to integrate specific strategies within and beyond the classroom, to increase physical activity and decrease sitting time across the school day—as future teachers<br>Perceived competence to effectively integrate specific active teaching strategies across the school day—as future teachers<br>Attitudes<br>Feelings about the impact of increasing activity and breaking sitting time on student outcomes (i.e. on task time, interest, academic outcomes)
Louth, 2015	Australia	Elementary school teachers<br>N = 7<br>57% female<br>86% over 40 yrs	Not reported	Single group pre-post<br>In-person (school)<br>6 months	***Training/professional development***<br>Professional development for teachers aimed to facilitate a change in the level of knowledge and expertise of teachers to embed regular physical activity through traditional indigenous games. In an initial professional development workshop teachers played the games and identified the safe and inclusive strategies they could use to introduce traditional indigenous games to their students.	Perceived abilities<br>-Confidence in teaching physical activity Knowledge<br>-Teacher knowledge of PA

Author	Country	Sample^	Theoretical framework	Study design, setting and duration	Capacity building strategies	Outcomes Measured
					Communities of practice<br>Teachers worked in groups to teach traditional indigenous games to their peers and in doing so gained real world experience facilitating these games to others.	
Mazzucca, 2017	United States	ECEs<br>N = 26<br>100% female<br>M age = 38.35	Social Cognitive Theory and Self-Determination Theory	RCT<br>In-person (childcare centres)<br>10 weeks	***Training/professional development***<br>Teachers attended two in-person, half-day workshop, which presented information about children's physical activity and sedentary behaviour at child care centres. Teachers learned about their role in promoting PA, opportunities to integrate physical activity and reduce sedentary time into their daily schedule, and teacher practices that can support physical activity and discourage sedentary behaviour.<br>***Communities of practice***<br>Teachers engaged in group discussions on how they can integrate what they learnt into their classroom and what motivated them to promote PA.<br>***Continued support***<br>Teachers were sent newsletters with guidance, reminded to set and monitor goals, received technical assistance through phone calls, emails and text messages.	<u>Perceived capabilities</u><br>-Self-efficacy for promoting physical activity and reducing SB<br><u>Attitudes</u><br>-Outcome expectations of having children be physically active in their classroom
Murtha, 2020	Australia	ECEs<br>N = 67<br>M age = NR<br>% Female NR	Social Cognitive Theory and Ecological Systems Theory	Single group pre-post<br>6 months<br>Online & in-person (childcare centres)	***Training/professional development***<br>The standard LEAPS professional development program required early childhood educators to complete 3 training modules, two of which were completed online and one which was completed face-to-face. The training covered multiple health topics including physical activity and active play.	<u>Perceived capabilities</u><br>Confidence in talking with families about PA<br>Confidence with running structured physical activity with children<br><u>Knowledge</u><br>What is the maximum number of hours of screen time that children aged 2-5 y should be watching each day?
Sevimli-Celik, 2021	Turkey	Preservice ECEs<br>N = 42<br>M = 21.3 yrs	Not reported	Single group pre-post<br>16 weeks	***Training/professional development***<br>Training consisted of four-hour weekly sessions for 15 weeks followed by final exam. The four-hour sessions were split into two-hours of theory and two-hours of practice. In the practice sessions, the course	<u>Perceived capabilities</u><br>Do you feel competent to teach PE to young children?<br><u>Knowledge</u>

Author	Country	Sample^	Theoretical framework	Study design, setting and duration	Capacity building strategies	Outcomes Measured
		100% female		In-person (university campus)	instructor, two teaching assistants, and the pre-service teachers met in the university gym to practice the ideas discussed in the classroom.<br>***Communities of practice***<br>In the second half of the semester, each student had an opportunity to teach a PE lesson to their peers in the university gym, revise the lesson, and repeat it in the university kindergarten with a group of children.	Do you feel competent about your PE subject knowledge?
Unlu, 2019	Turkey	Pre-service teachers<br>N = 56<br>M = 20.36 yrs<br>50% Female	Not reported	Non-randomized controlled trial<br>In-person (university campus)<br>1 week	***Training/professional development***<br>Pre-service teachers engaged in the Physical Education Science Counselling for Classroom Teachers course. Theoretical and practical knowledge and skills were taught by experts in the field.	Perceived capabilities<br>The Physical Education Teaching Efficacy scale [6]
Ward, 2020	Canada	ECEs<br>N = 204<br>41.7% < 30 yrs<br>24.7 30-39 yrs<br>30.2 40+ yrs<br>% Female NR	Socio-Ecological Model	Cluster RCT (two intervention groups)<br>Online & in-person (childcare centres)<br>9 months	**In person training group**<br>***Training/professional development***<br>3-hour training session during which experts discussed the importance of physical activity for young children, how to build FMS, and how to incorporate physical activity into their daily routine.<br>***Resources***<br>Participants' centres were also given an evidence informed resource pack that included activity ideas and information to help educators provide opportunities for children to be physically active.<br>***Continued support***<br>On-going support was provided via phone or email once or twice a month by the provincial coordinator and a 90-minute on site "Booster session" took place three to six months after the in-person training<br>**Online training group**<br>***Training/professional development***<br>Online training consisted of two modules, one of which focused on PA. The online training took about four-hours to complete and covered the same topics as the in-person training. Videos of best practices were filmed in a local childcare centre and embedded in the modules to ensure the training was visually interesting and interactive.	Knowledge<br>FMS skills and physical activity knowledge

Author	Country	Sample^	Theoretical framework	Study design, setting and duration	Capacity building strategies	Outcomes Measured
					Resources<br>Participants were sent an Active Kids Toolkit.<br>***Continued support***<br>On-going support was provided via phone or email by the provincial coordinator and participants were offered access to webinars.	
Webster, 2011	United States	Pre-service classroom teachers (elementary and early childhood)<br>N = 201<br>M = 20.8 yrs<br>96% females	Not reported	Single group pre-post<br>In-person (university campus)<br>16 weeks	***Training/professional development***<br>Pre-service teachers participated in a physical activity promotion course. Instructional strategies/learning experiences included dyadic instruction in the classroom related to major physical activity concepts, physical activity recommendations benefits of children engaging in PA, trends in children's PA.<br>***Communities of practice***<br>Pre-service teachers were given the opportunity to practice what they learnt to their peers.	<u>Perceived capabilities</u><br>School-Physical Activity Promotion Competence Questionnaire [7]<br><u>Attitudes</u><br>School-Physical Activity Promotion Attitudes Questionnaire [7]
Whipp, 2011	Australia	Elementary school teachers<br>N = 5<br>M age = 33 yrs<br>40% Female	Not reported	Single group pre-post<br>In-person (schools)<br>6 months	***Mentor***<br>Sixty minute PE sessions were delivered by external specialists. All sessions consisted of four components: warm-ups, coordination and agility, skills and games, and core movement. Generalist teachers attended all PE sessions delivered by the external specialists, and were instructed to observe and ask any questions they wished before, during, or after the sessions.	<u>Perceived capabilities</u><br>Teacher's perceptions of their ability to provide students with PE requirements. Items included: inclusivity, different abilities, safety, FMS, and curriculum framework outcomes.
Wright, 2020	Canada	Elementary teachers<br>N = 23<br>87% female<br>25-44 years	Cooperative Framework	Single group pre-post<br>In-person (university campus)<br>10 weeks	**Mentor**<br>Two experts in physical literacy ran physical education sessions, one who facilitated the activities, and the other who engaged the teacher in observation and discussion relating to the sessions. The content of the sessions consisted of games and activities that developed competence in movement skills and built confidence, motivation and knowledge of physical activity in the children. Teachers were also encouraged to engage in the activities in order to gain a greater understanding.<br>***Resources***<br>Teachers were provided with online resources to supplement the in-class activities.	<u>Perceived capability</u><br>Total confidence relating to engage children in physical activity and play

Author	Country	Sample^	Theoretical framework	Study design, setting and duration	Capacity building strategies	Outcomes Measured
Xiang, 2002	United States	Pre-service elementary teachers<br>N = 97<br>M = 20.82 yrs<br>94.8% Female	Not reported	Single group pre-post<br>In-person (university campus)<br>15 weeks	***Training/professional development***<br>Physical education methods course consisting of 3-hour lecture on campus and 2-hour lab in local public school once a week. Lectures sessions included hearing lectures, watching videos, and participating in activities that reinforced the course material both in a classroom and a gymnasium.<br>***Mentor***<br>Students attended a local public school once a week for 10 weeks as an assistant to a mentor physical education teacher. The participants helped the children individually with skills and activities, led exercises, supervised games, and assisted the teacher.	Attitudes<br>Positive and negative values relating to elementary PE

1. Callea, M. B., Spittle, M., O'Meara, J., & Casey, M. (2008). Primary school teacher perceived self-efficacy to teach fundamental motor skills. *Research in Education*, *79*(1), 67-75.
2. Centeio EE, Barcelona J, Moore EWG, McKown H, Erwin H. (2020) Elementary and Secondary Classroom Teachers' Efficacy for Providing Physical Activity Breaks. Accepted for roundtable presentation at the 2020 American Educational Research Association International Conference, San Francisco, CA. Conference canceled due to COVID-19; 2020
3. Bruijns, B. A., Johnson, A. M., Burke, S. M., & Tucker, P. (2022). Educators' Self-Efficacy to Promote Physical Activity and Outdoor Play and Minimize Sedentary Behaviors in Childcare: A Tool Validation Study. *Journal of Research in Childhood Education*, 1-10.
4. Bruijns, B. A., Johnson, A. M., Burke, S. M., & Tucker, P. (2022). Validation of a physical activity, sedentary behavior, and outdoor play behavioral intention and perceived behavioral control tool for early childhood educators. *Early Childhood Education Journal*, 1-9.
5. Derscheid, L. E., Kim, S. Y., Zittel, L. L., Umoren, J., & Henry, B. W. (2014). Teachers' self-efficacy and knowledge of healthy nutrition and physical activity practices for preschoolers: instrument development and validation. *Journal of Research in Childhood Education*, *28*(2), 261-276.
6. Humphries, C. A., Hebert, E., Daigle, K., & Martin, J. (2012). Development of a physical education teaching efficacy scale. *Measurement in Physical Education and Exercise Science*, *16*(4), 284-299.
7. Webster, C., Monsma, E., & Erwin, H. (2010). The role of biographical characteristics in preservice classroom teachers' school physical activity promotion attitudes. *Journal of Teaching in Physical Education*, *29*(4), 358-377.
