# Supplementary material for: Effect of capacity building interventions on classroom teacher and early childhood educator perceived capabilities, knowledge, and attitudes relating to physical activity and fundamental movement skills: a systematic review and meta-analysis
Source: BMC Public Health. 2024 May 27;24:1409. doi: 10.1186/s12889-024-18907-x (PMC11129429; doi:10.1186/s12889-024-18907-x)
Supplement: Supplementary file 3 — Supplementary Material 3 [file 12889_2024_18907_MOESM3_ESM.pdf]

Appendix E – Funnel plots from meta-analyses

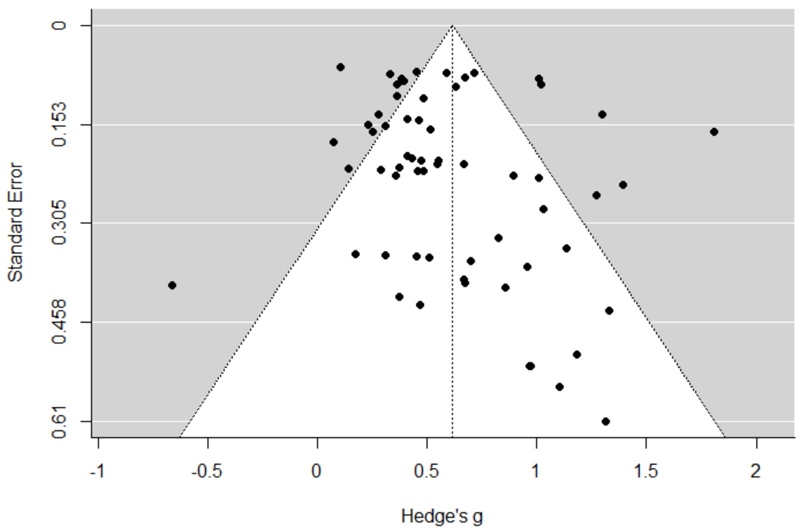

Figure E1 – Funnel plot of effect sizes and standard errors for perceived capacity

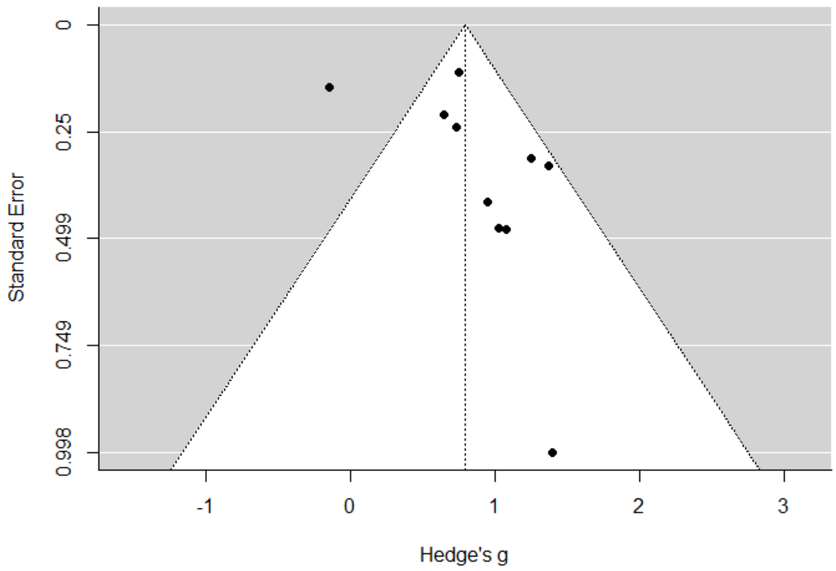

Figure E2 – Funnel plot of effect sizes and standard errors for knowledge

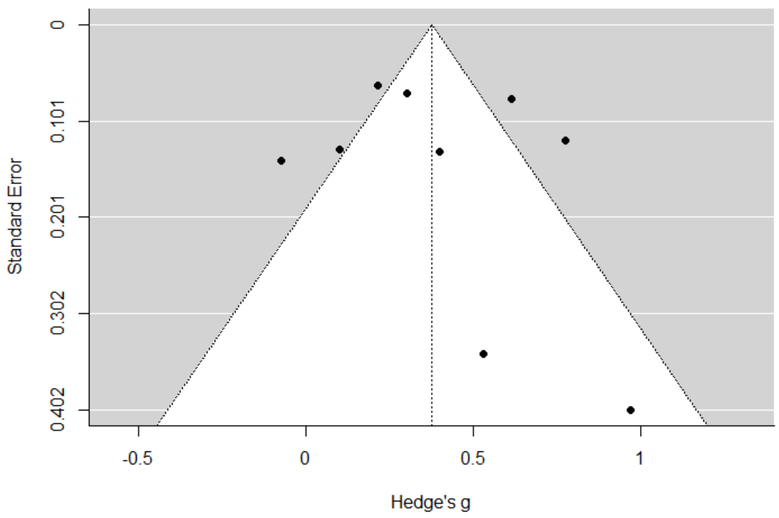

Figure E3 – Funnel plot of effect sizes and standard errors for attitudes
