# Supplementary material for: Effect of capacity building interventions on classroom teacher and early childhood educator perceived capabilities, knowledge, and attitudes relating to physical activity and fundamental movement skills: a systematic review and meta-analysis
Source: BMC Public Health. 2024 May 27;24:1409. doi: 10.1186/s12889-024-18907-x (PMC11129429; doi:10.1186/s12889-024-18907-x)
Supplement: Supplementary file 4 — Supplementary Material 4 [file 12889_2024_18907_MOESM4_ESM.pdf]

	R	D	Mi	Me	S	O
Bruijns et al. 2021	+	+	?	?	+	?
Duff et al. 2019	−	+	?	?	?	−
Hivner et al. 2019	−	+	?	?	−	−
Hofmann et al. 2020	+	+	+	+	?	?
Mazzucca et al. 2017	?	+	+	?	?	?
Ward et al. 2020	+	+	+	?	?	?

Figure D1 – Risk of bias for randomized controlled trials
R = Bias arising from the randomization process
D = Bias due to deviations from intended intervention
Mi = Bias due to missing data
Me = Bias due to measurement of the outcome
S = Bias in selection of the reported results
O = Overall risk of bias

+ = Low risk

? = Some concerns

− = High risk

	Co	P	Cl	D	Mi	Me	S	O
Altunsöz et al. 2015	−	+	?	+	+	−	?	−
Unlu 2019	+	−	+	+	+	−	?	−

Figure D2 – Risk of bias for non-randomized controlled trials
Co = Risk of bias due to confounding
P = Bias in selection of participants into the study
Cl = Bias in classification of the intervention
D = Bias due to deviations from intended intervention
Mi = Bias due to missing data
Me = Bias due to measurement of the outcome
S = Bias in selection of the reported results
O = Overall risk of bias

\+ = Low risk

? = Some concerns

− = High risk

	1	2	3	4	5	6	7	8	9	10	11	12	Overall
Bai et al. 2019	Y	Y	Y	NR	Y	Y	N	N	N	Y	N	Y	Fair
Barcelona et al. 2022	Y	N	NR	NR	Y	Y	Y	N	Y	Y	N	Y	Fair
Bruijns et al. 2022	Y	Y	Y	NR	Y	Y	Y	N	Y	Y	N	NA	Good
Fletcher et al. 2013	N	N	Y	NR	Y	Y	N	N	Y	Y	N	Y	Fair
Johnson-Shelton et al. 2022	Y	N	Y	NR	Y	Y	N	N	Y	Y	N	Y	Fair
Lander et al. 2019	Y	Y	Y	N	Y	Y	N	N	Y	Y	N	Y	Good
Lander et al. 2020	N	Y	Y	N	Y	Y	N	N	Y	Y	N	Y	Good
Louth et al. 2015	N	N	Y	NR	N	Y	N	N	Y	N	N	NA	Poor
Murtha et al. 2020	N	N	NR	NR	Y	N	N	N	Y	N	N	NA	Poor
Sevimli-Celik et al. 2021	Y	N	Y	NR	Y	Y	N	N	Y	N	N	NA	Poor
Webster et al. 2011	Y	N	Y	NR	Y	Y	Y	N	Y	Y	N	Y	Good
Whipp et al. 2011	Y	N	Y	NR	N	N	N	N	Y	Y	N	NA	Poor
Wright et al. 2020	Y	N	Y	N	N	Y	N	N	Y	Y	N	Y	Fair
Xiang et al. 2002	Y	N	Y	NR	Y	Y	Y	N	Y	Y	N	Y	Fair

Figure D3 – Risk-of-bias of single group pre-post studies

1 = Was the study question or objective clearly stated? 2 = Were eligibility/selection criteria for the study population prespecified and clearly described? 3 = Were the participants in the study representative of those who would be eligible for the test/service/intervention in the general or clinical population of interest? 4 = Were all eligible participants that met the prespecified entry criteria enrolled? 5 = Was the sample size sufficenty large to provide confidence in the findings? 6 = Was the test/service/intervention clearly described and delivered consistently across the study population? 7 = Were the outcome measures prespecified, clearly defined, valid, reliable, and assessed consistently across all study participants? 8 = Were the people assessing the outcomes blinded to the participants' exposures/interventions? 9 = Was the loss to follow-up after baseline 20% or less? Were those lost to follow-up accounted for in the analysis? 10 = Did the statistical methods examine changes in outcome measures from before to after the intervention? Were statistical tests done that provided p values for the pre-to-post changes? 11 = Were outcome measures of interest taken multiple times before the intervention and multiple times after the intervention (i.e., did they use an interrupted time-series design)? 12 = If the intervention was conducted at a group level (e.g., a whole hospital, a community, etc.) did the statistical analysis take into account the use of individual-level data to determine effects at the group level? CD = could not determine, N = no, NA = not applicable NR = not reported, Y = yes.
